# Public Database-Driven Insights Into Aging Stress-Associated Defective Gut Barrier With Low SARS-CoV-2 Receptors

**DOI:** 10.3389/fmed.2020.606991

**Published:** 2020-12-22

**Authors:** Yuseok Moon

**Affiliations:** ^1^Laboratory of Mucosal Exposome and Biomodulation, Department of Convergence Medical Sciences, Pusan National University, Yangsan, South Korea; ^2^Graduate Program of Genome Data Sciences, Pusan National University, Yangsan, South Korea

**Keywords:** SARS-CoV-2, gut barrier, integrated stress responses, metabolic stress, aging

## Abstract

The novel coronavirus disease (COVID-19), caused by the severe acute respiratory syndrome coronavirus 2 (SARS-CoV-2), has led to a global pandemic, and resulted in high case-fatality rate in the elderly. In addition to typical respiratory responses, ~50% of clinical cases include gastrointestinal symptoms such as diarrhea, vomiting, abdominal pain, and persistent fecal shedding of the virus even after its clearance from the pulmonary system. In the present study, we assessed aging-associated gut transcriptomic responses considering the gastrointestinal symptoms contributing to COVID-19 severity. Intestinal expression of SARS-CoV-2 receptors and defense biomarkers decreased with increasing age. Moreover, aging-associated integrated stress responses (ISR) and mTOR-linked cell metabolic stress signals counteracted gut defense biomarkers. However, SARS-CoV-2 receptor expression was positively associated with gut barrier integrity potently via downregulation of the two stress-responsive signals. Gut transcriptome-based mechanistic prediction implicates that high susceptibility to COVID-19 in the elderly with low SARS-CoV-2 receptors is due to aging stress-associated defective gut defense, providing a new avenue for viral entry receptor-independent interventions.

## Introduction

Since the first report on an unknown pneumonia-like disorder caused by the severe acute respiratory syndrome coronavirus-2 (SARS-CoV-2) in Wuhan area, the coronavirus disease (COVID-19) has become a worldwide pandemic, and is majorly attributed to zoonotic sources (1–3). Although the common symptoms include fever, cough, dyspnea, fatigue, and sputum production, fatal cases present lymphopenia and severe inflammatory distress such as organ failure in addition to airway dysfunction (4). Such severe complications are prominent in subjects with underlying health conditions including cardiovascular diseases, diabetes, or obesity, requiring hospitalization and intensive care (4–7). Moreover, based on the population-based studies, the elderly group (particularly aged 70 years or older) among the patients with COVID-19 presented high case-fatality rate with severe complications in Italy and China (3, 8, 9). A quantitative systemic review demonstrated that ~25 and 71% of the elderly subjects developed renal injuries and required supplementary oxygen, respectively (8). Although the complications of COVID-19 with aging are evident, its mechanistic assessments are required for developing precise interventions for the susceptible population.

During cellular infection by SARS-CoV-2, the viral spike (S) protein recognizes angiotensin converting enzyme 2 (ACE2) as a viral receptor to enter the host cells. Moreover, this entry requires S protein priming by cellular proteases, which entails S protein cleavage and allows fusion of viral and cellular membranes. SARS-CoV-2 employs the cellular serine protease, transmembrane protease serine 2 (TMPRSS2), which cleaves the S protein of human coronaviruses on the cell membrane for priming (10). Successful viral entry depends on ACE2 and TMPRSS2, which are not only coexpressed in the airway epithelia but also highly expressed in gut cells such as esophageal, ileal, and colonic epithelial cells (11), indicating that the gastrointestinal tract acts as an alternative route for SARS-CoV-2 invasion. Furthermore, for ~50% of COVID-19 clinical cases, SARS-CoV-2 can be detected in fecal samples and gut mucosa of the infected hosts (12–14). In addition, half of infected patients display prolonged fecal shedding of SARS-CoV-2 even after viral clearance from the respiratory tract (15), thereby suggesting the transmission of coronavirus via fecal–oral route. In particular, persistent inflammatory distress in the insulted gut during viral infection may contribute to COVID-19 severity.

In response to viral infection, human cells activate a common adaptive pathway, known as the integrated stress response (ISR), to restore cellular integrity. The core biochemical event in ISR is the phosphorylation of eukaryotic translation initiation factor 2 alpha (eIF2α) by the eIF2α kinase family, leading to global translational arrest, and the induction of specific stress-responsive genes to achieve biological homeostasis in the insulted hosts (16, 17). In the present study, assuming age to be a crucial risk factor of COVID-19 severity, we investigated the transcriptomic features of human gut with aging stress. In particular, the aging stress in association with ISR and other stress signaling was evaluated to predict the defective responses to SARS-CoV-2 in the elderly subjects.

## Methods

### Age-Linked Transcriptome Data

RNA-seq raw counts and normalized TPM matrices (Illumina paired-end, 76 bp) were downloaded from the Genotype-Tissue Expression (GTEx) Portal (version 8, 17,382 samples from 30 tissue types). All accessed data used in this study are publicly available on the web portal (https://gtexportal.org/home/index.html) and have been deidentified, except for patient age range and gender. Non-diseased transverse colon tissues (*n* = 937) containing the mucosal parts from the different age groups were selected for the transcriptomic analysis (Supplementary Figure 1A). Samples from the sigmoid colon without the mucosa were excluded.

### Genomic Analysis Using Colon Cancer Datasets

Clinical sources of transcriptomic data from colon cancer tissue samples of patients are listed in the dataset (GEO ID: gse39582, *n* = 566). Among a large series of colon cancer data collected for the Cartes dIdentité des Tumeurs (CIT) program from the French Ligue Nationale Contre le Cancer (http://cit.ligue-cancer.net), 566 were analyzed for mRNA expression profiles using Affymetrix U133plus2 chip and, among these, 463 were analyzed for DNA alteration profiles using the CGH Array (CIT-CGHarray V6). Survival analysis was performed in three datasets of patients with colorectal cancer (gse39582 [*n* = 566], gse24551 [*n* = 333], and gse14333 [*n* = 290]). Dataset gse24551 was derived from genome-wide expression at exon level for two independent series of colorectal cancer tissue biopsies using the Affymetrix Human Exon 1.0 ST platform. Dataset gse14333 was from the expression profiles of surgically resected specimens in 290 patients with colorectal cancer using Affymetrix Human Genome U133Plus 2.0 arrays.

### Genomic Analysis Using IBD Datasets

Human intestinal tissue datasets were obtained from the gene expression arrays of patients with IBD (gse117993, *n* = 190). These experiments tested the differential gene expression in these three types of IBD relative to healthy control samples. RNA was isolated from biopsies from 190 pediatric patients undergoing diagnostic colonoscopy for inflammatory bowel diseases, including Crohn's disease (CD) and ulcerative colitis (UC). Single-end, 75-bp sequencing was performed, and raw reads were aligned to the human genome using Gencode v 24 as a reference. We included 14,085 protein-coding mRNA genes in downstream analyses. For clinical dataset, the three major clinical subsets of IBD included only UC, colon-only CD (cCD), and ileocolonic CD (iCD) (Supplementary Figure 1B).

### Statistics

Statistical analyses were performed using GraphPad Prism v. 5.01 (La Jolla, CA, USA). For comparative analysis of two groups of data, Student's *t*-test was performed. For comparative analysis of multiple groups, data were subjected to analysis of variance (ANOVA) with *Newman–Keuls* method as a *post-hoc* ANOVA assessment.

## Results

### Aging Attenuates Expression of SARS-CoV-2 Receptors and Gut Defense Biomarkers

**Figure 1:**
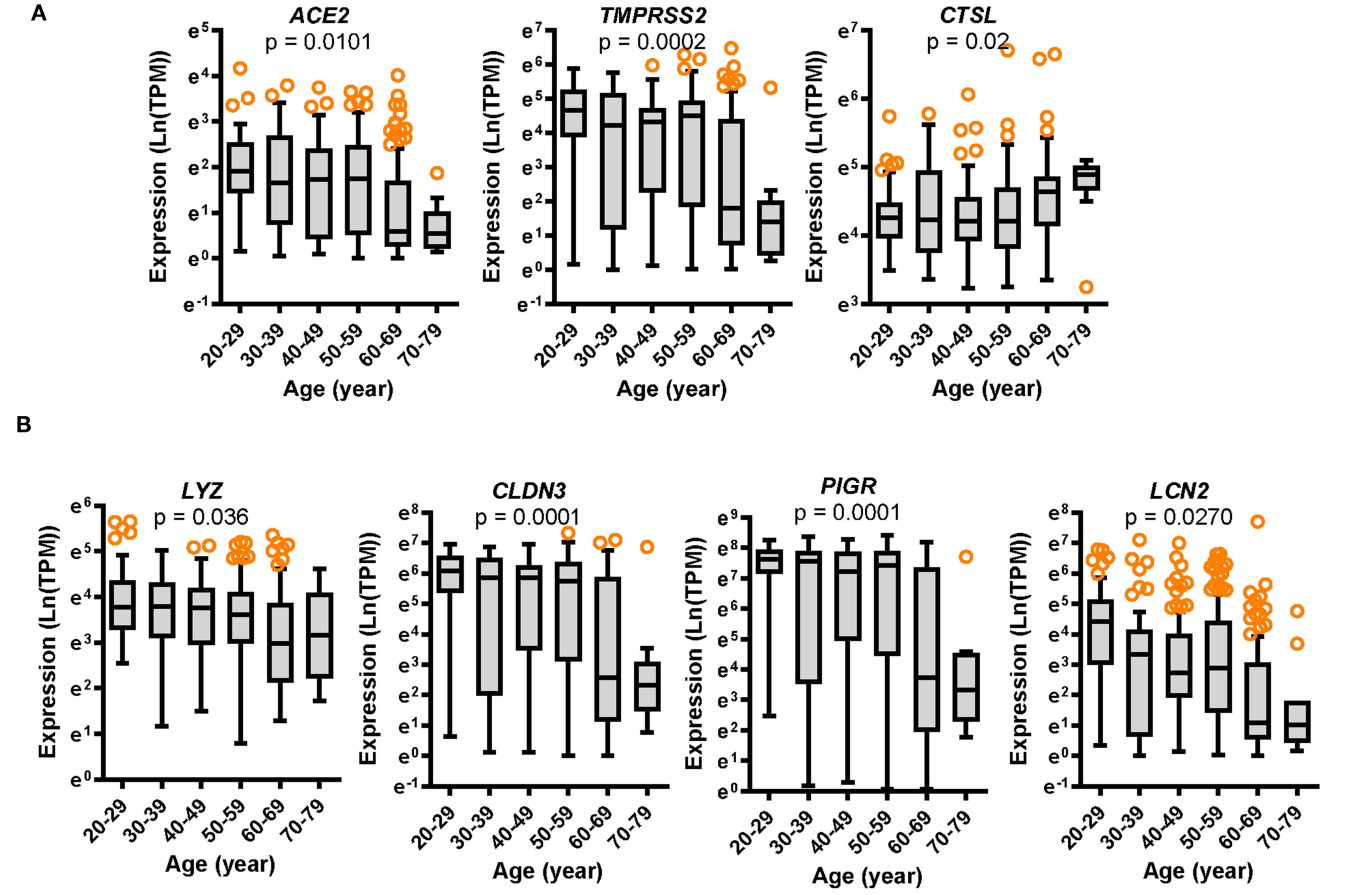
Expression of SARS-CoV-2 receptors and gut defense biomarkers with age. Results are depicted as box-and-whisker plots (Turkey) for the expression of SARS-CoV-2 receptors *ACE2, TMPRSS2*, and *CTSL* **(A)** or gut defense makers *LYZ, CLDN3, PIGR*, and *LCN2* **(B)** in normal mucosal intestinal tissues (GTEx dataset v8). Values are presented as natural logarithm of transcripts per million (TPM). Statistical significance of the expression variation with age is illustrated on the top of each plot (Kruskal–Wallis test).

We analyzed aging-associated patterns mainly using the transcriptome dataset of non-diseased tissue from the Genotype-Tissue Expression (GTEx) project. Since the gut is a persistent source of fecal SARS-CoV-2 production, we specifically analyzed colonic RNA-seq transcriptomes from donors of varying ages (aged 20–79 years) (Figures 1A,B). Expression of two SARS-CoV-2 receptors was assessed in different age groups. Compared to the levels in young age group (aged 20–29 years), expression of *ACE2* and *TMPRSS2* tended to decrease with age, which was prominent in elderly groups (aged 60–79 years) (Figure 1A). For successful viral entry by directly binding to ACE2, other accessary components such as TMPRSS2 and cathepsin L (CTSL) can facilitate S protein priming for receptor binding on the host cell surface (10); however, CTSL without proteolysis activity on the cell surface is dispensable for host cell entry of SARS-CoV-2 (10, 18). In contrast with the levels of *ACE2* and *TMPRSS2, CTSL* expression tended to increase with age in the colon tissue (Figure 1A).

### SARS-CoV-2 Receptors Are Positively Associated With Gut Defense During Aging or Chronic Disease Progression

**Figure 2:**
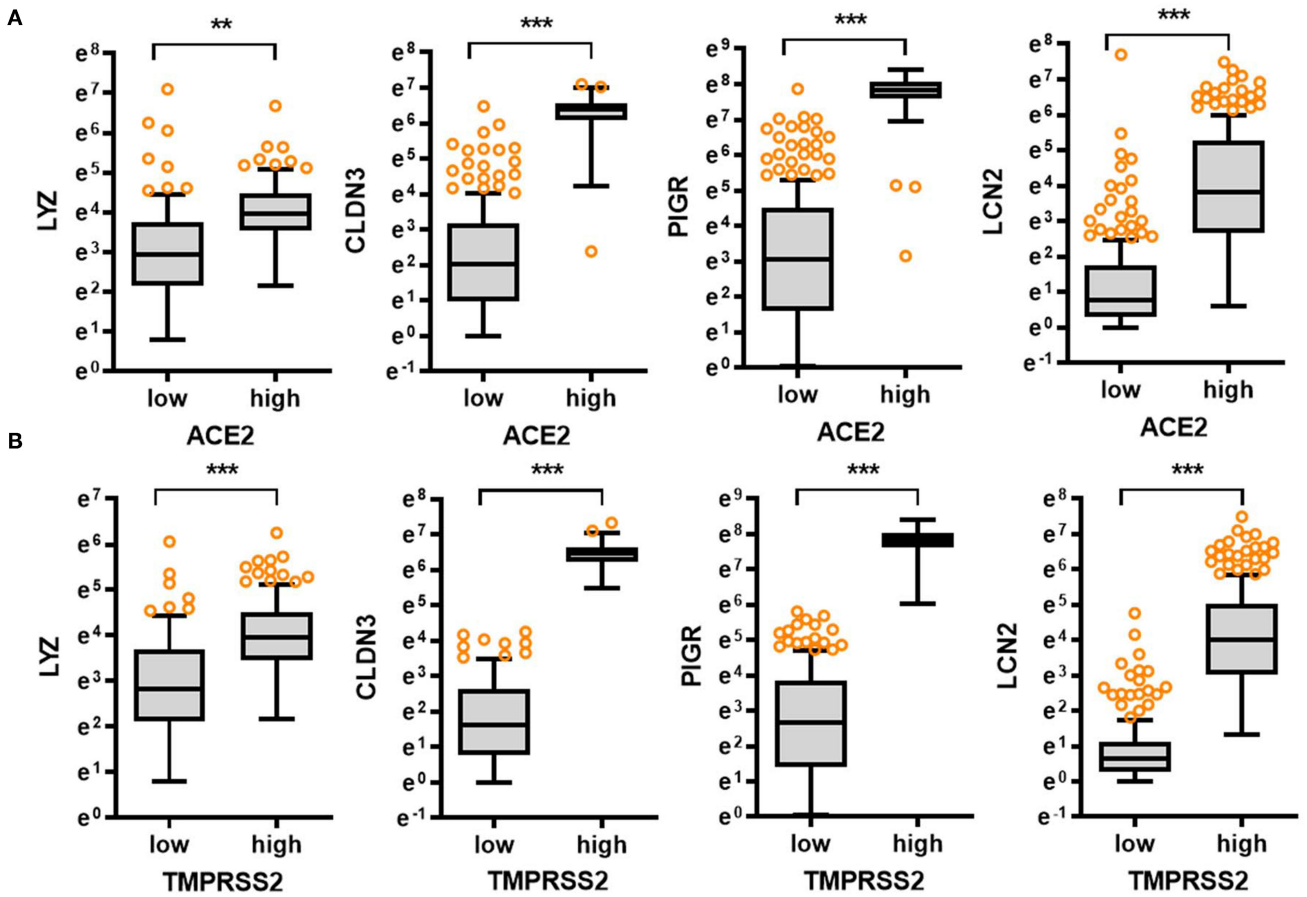
Comparative expression of barrier defense biomarkers with SARS-CoV-2 receptor levels. Based on *ACE2* **(A)** or *TMPRSS2* **(B)** levels in the normal mucosal intestinal tissues (GTEx dataset v8), we selected the 150 highest and 150 lowest level samples, which were further evaluated based on *LYZ, CLDN3, PIGR*, and *LCN2* levels. Values are presented as natural logarithm of transcripts per million (TPM). Asterisks (*) indicate significant differences from the low expression group (**p < 0.01, ***p < 0.001).

In response to viral entry, the host epithelial defense is a deterministic factor of the pathogenic outcomes in infected patients. We analyzed the expression of gut barrier defense biomarkers such as lysozyme (*LYZ*), claudin 3 (*CLDN3*), polymeric immunoglobulin receptor (*PIGR*), and lipocalin 2 (*LCN2*) in the colon. Expression of the corresponding genes *LYZ, CLDN3, PIGR*, and *LCN2* was modestly associated with age (*p* = 0.036, *p* = 0.0001, *p* = 0.0001, and *p* = 0.0270, respectively) and tended to decrease with increasing age (Figure 1B). Notably, levels of gut defense biomarkers were significantly attenuated in the elderly subjects (aged 60–79 years). Moreover, expression of key SARS-CoV-2 receptors was positively associated with levels of gut defense biomarkers (Figures 2A,B). From the GTEx-based dataset, subjects with high expression of *ACE2* or TMPRSS2 presented high levels of *LYZ, CLDN3, PIGR*, and *LCN2* in the intestine, indicating a protective action of SARS-CoV-2 receptors against gut infection.

**Figure 3:**
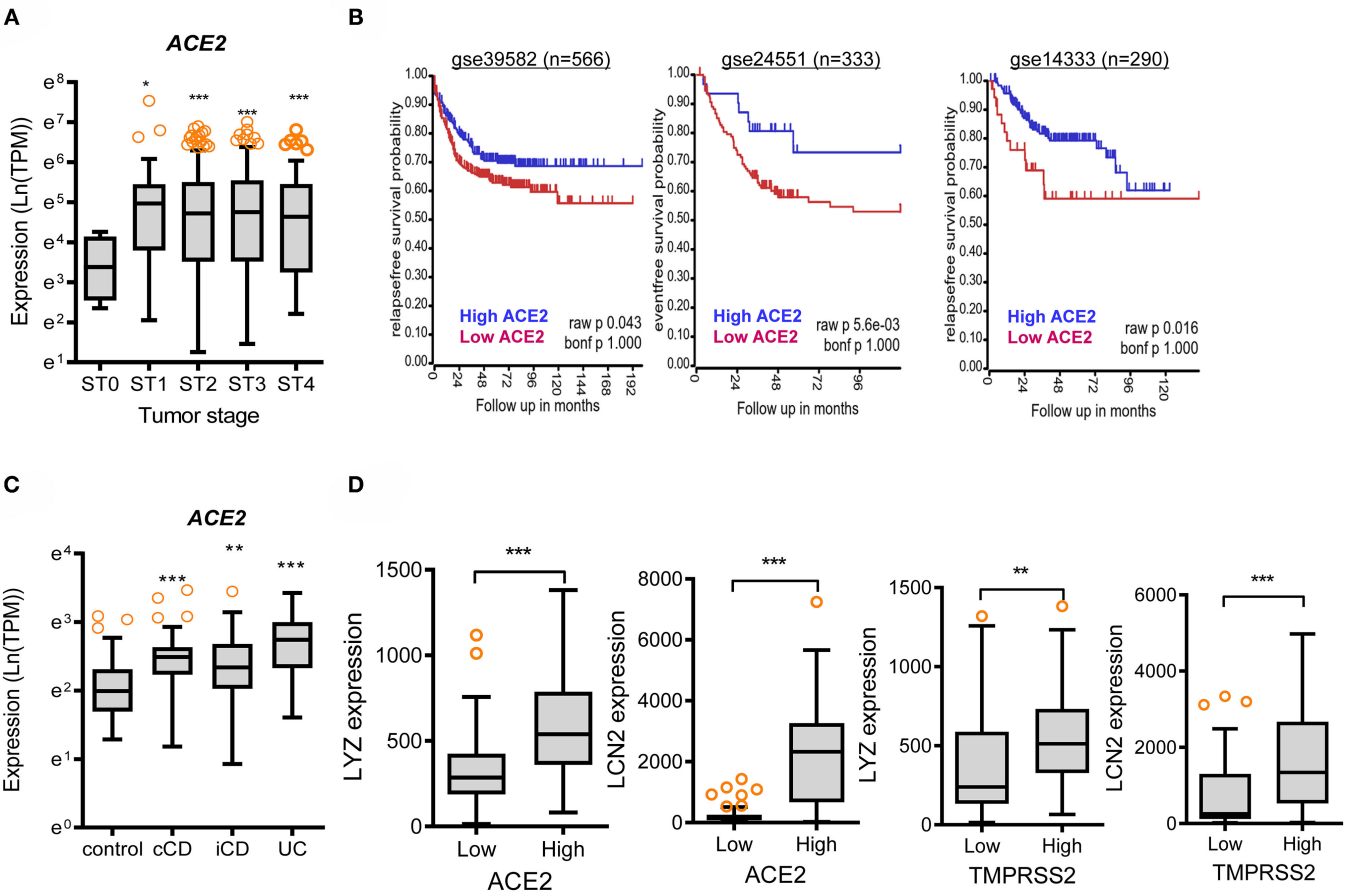
Involvement of SARS-CoV-2 receptors in chronic intestinal diseases. **(A)** *ACE2* expression in different tumor stages from the transcriptome dataset in patients with colon cancer (GEO ID: gse39582, *n* = 566). Values are presented as natural logarithm of transcripts per million (TPM). Asterisks (*) indicate significant differences from levels at Stage 0 (*p < 0.05, **p < 0.01, ***p < 0.001 using two-tailed unpaired Student's *t-*test). **(B)** Kaplan–Meier plot of survival analysis based on tissue *ACE2* transcript levels in patients with CRC from three datasets (gse39582 [*n* = 566, expression cutoff 150.5], gse24551 [*n* = 333, expression cutoff 444.6], and gse14333 [*n* = 290, expression cutoff 44.5]). **(C)** Intestinal expression of *ACE2* was compared in patients with different IBD types from datasets gse117993 (*n* = 190). UC, ulcerative colitis; cCD, colon-only CD; iCD, ileocolonic CD. Values are presented as natural logarithm of transcripts per million (TPM). Results are depicted as box-and-whisker plots (Turkey). Asterisks (*) indicate significant differences from the control group (*p < 0.05, **p < 0.01, ***p < 0.001 using two-tailed unpaired Student's *t-*test). **(D)** Based on *ACE2* or *TMPRSS2* levels from datasets gse117993 (*n* = 190), we selected the 150 highest and 150 lowest level samples, which were further evaluated based on *LYZ* and *LCN2* levels. Values are presented as natural logarithm of TPM. Asterisks (*) indicate significant differences from the low expression group (*p < 0.05, **p < 0.01, ***p < 0.001).

In addition to the analyses of the non-diseased tissues from GTEx project, gene expression in biopsies from patients with chronic intestinal distress [colon cancer and inflammatory bowel disease (IBD)] was also evaluated. In patients with colon cancer, *ACE2* expression tended to increase with disease progression (Figure 3A), whereas *TMPRSS2* levels were not significantly altered in the lesions (Supplementary Figure 2A); however, patients with high levels of *ACE2* displayed good prognosis compared to those with low expression (Figure 3B). Moreover, high expression levels of *TMPRSS2* are positively associated with good prognoses for CRC patients (Supplementary Figure 2B). Results of survival analyses demonstrate the protective roles of SARS-CoV-2 receptors in oncological disease progress, which were in accordance with the results in non-diseased colonic tissues of the GTEx dataset. Furthermore, the patterns were also verified in the tissue expression from patients with IBD (Figures 3C,D). Expression of *ACE2* and *TMPRSS2* was markedly elevated in patients with UC and CD, including colon-only CD (cCD) and ileocolonic CD (iCD), when compared to that in the control group (Figure 3A and Supplementary Figure 2C, respectively). Patients with high expression of *ACE2* or *TMPRSS2* displayed high levels of *LYZ* and *LCN2* in the intestine (Figure 3D), indicating a protective action of SARS-CoV-2 receptors against gut barrier disruption.

### Aging-Associated ISR Potently Counteracts Levels of Gut Defense Biomarkers

**Figure 4:**
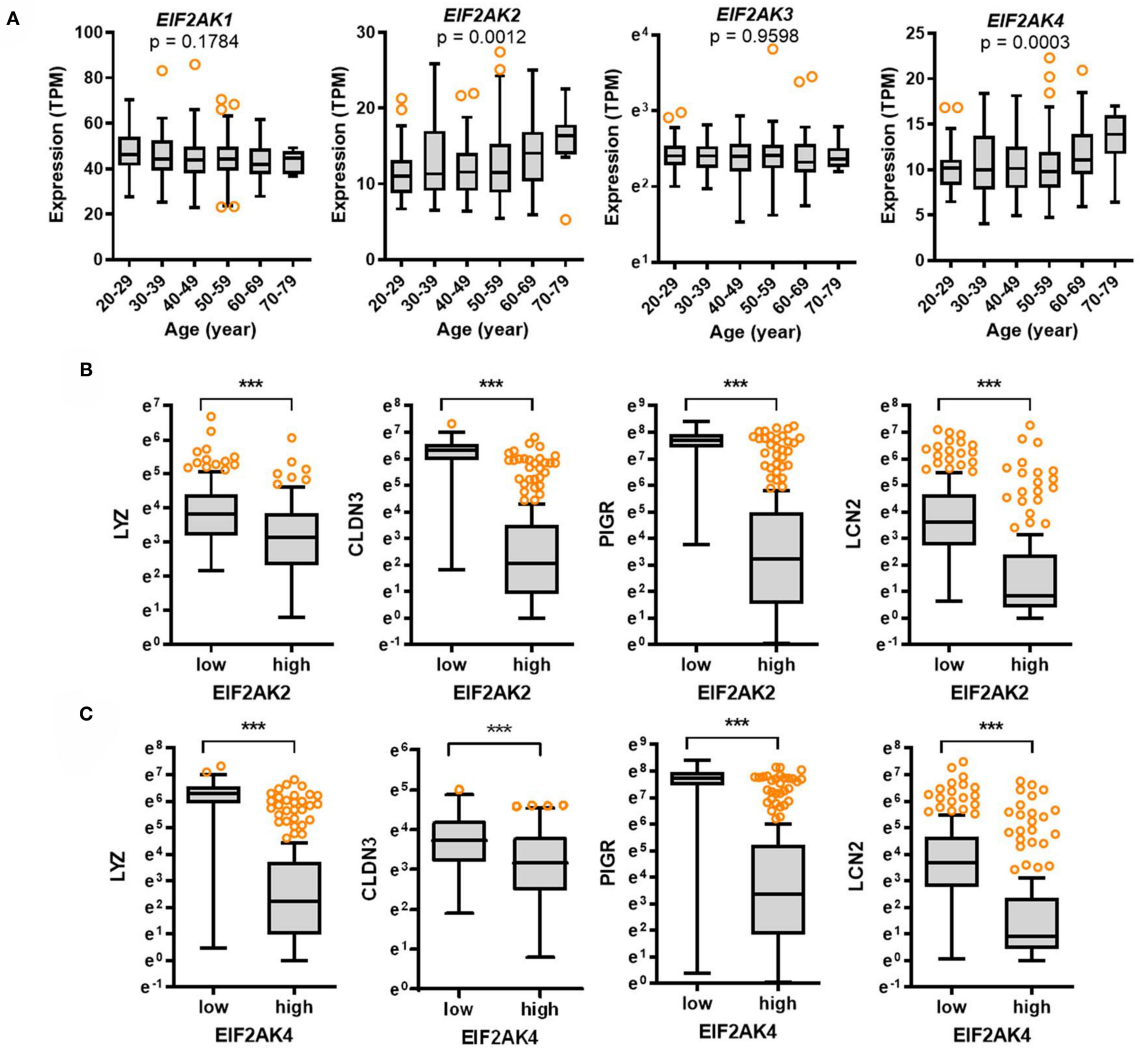
Expression of eIF2α kinases and their association with gut defense biomarkers with age. **(A)** Results are depicted as box-and-whisker plots (Turkey) for expression of eIF2α kinases (*EIF2AK1, EIF2AK2, EIF2AK3, or EIF2AK4*) in normal mucosal intestinal tissues (GTEx dataset v8). Values are presented as transcripts per million (TPM). Statistical significance of the expression variation with age is illustrated on the top of each plot (Kruskal–Wallis test). **(B,C)** Considering the *EIF2AK2* **(B)** or *EIF2AK4* **(C)** levels, we selected the 150 highest and 150 lowest level samples, which were further evaluated based on *LYZ, CLDN3, PIGR*, and *LCN2* levels. Values are presented as natural logarithm of TPM. Asterisks (*) indicate significant differences from the low expression group (***p < 0.001).

To elucidate the molecular mechanisms of gastrointestinal distress, eIF2α kinase-mediated ISR was evaluated as the common adaptive pathway in response to the external insults including viral infection. The alpha subunit of eIF2 is targeted by four different stress-related mammalian protein kinases, namely, heme-regulated eIF2α kinase (HRI, EIF2AK1), double-stranded RNA-dependent protein kinase R (PKR, EIF2AK2), RNA-dependent protein kinase-like ER kinase (PERK, EIF2AK3), and eIF2α kinase general control non-repressed 2 (GCN2, EIF2AK4) (16, 17). In particular, SARS-CoV-2-infected cells display a PKR-linked pathogenesis including specific 28S rRNA cleavage (19–21). Expression of four eIF2α kinases was assessed in different age groups. Expression of *EIF2AK2* and *EIF2AK4* was significantly associated with age (*p* = 0.0012, and *p* = 0.0003, respectively) and tended to increase with age (Figure 4A). Notably, the levels of *EIF2AK2* and *EIF2AK4* were significantly elevated in elderly subjects (aged 60–79 years) when compared to those in the young group (aged 20–29 years). Furthermore, we evaluated whether eIF2α kinases are involved in regulation of gut barrier integrity. Expression of *EIF2AK2* or *EIF2AK4* was positively associated with the levels of gut defense biomarkers (Figures 4B,C). Subjects with high expression of *EIF2AK2* or *EIF2AK4* displayed low levels of *LYZ, CLDN3, PIGR*, and *LCN2* in the intestine, thereby suggesting a negative regulation of gut defense by eIF2α kinase-linked signaling.

### Aging-Associated Cell Metabolic Stress Downregulates Levels of Gut Defense Biomarkers

**Figure 5:**
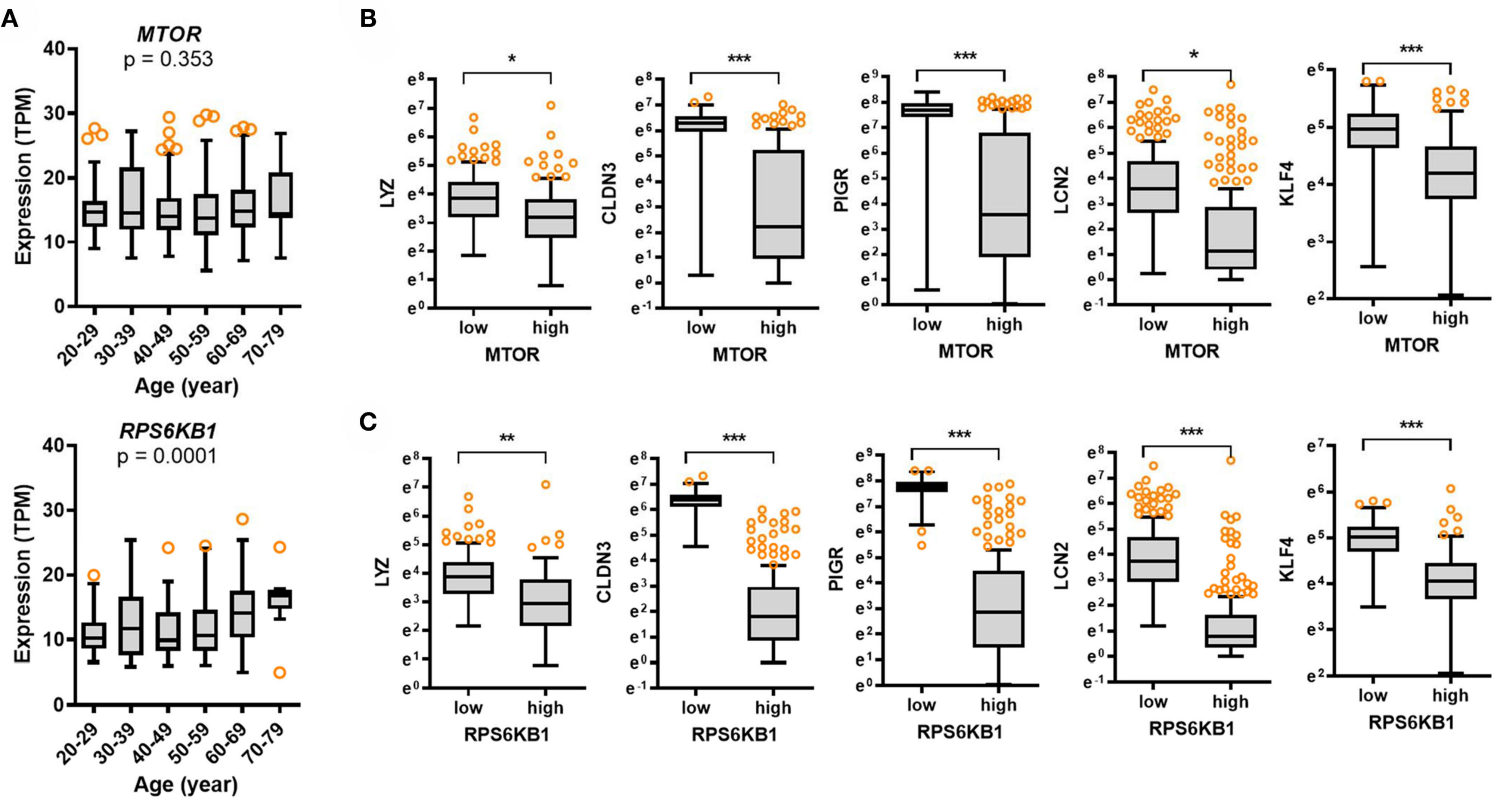
Expression of cell metabolic stress markers and their association with gut defense biomarkers with age. **(A)** Results are depicted as box-and-whisker plots (Turkey) for expression of cellular metabolic stress markers (*mTOR or RPS6KB1*) in normal mucosal intestinal tissues (GTEx dataset v8). Values are presented as transcripts per million (TPM). Statistical significance of the expression variation with age is illustrated on the top of each plot (Kruskal–Wallis test). **(B,C**) Considering the *mTOR* **(B)** or *RPS6KB1* **(C)** levels, we selected the 150 highest and 150 lowest level samples, which were further evaluated based on *LYZ, CLDN3, PIGR*, and *LCN2* levels. Values are presented as natural logarithm of TPM. Asterisks (*) indicate significant differences from the low expression group (*p < 0.05, **p < 0.01, ***p < 0.001).

The mammalian target of rapamycin (mTOR) is a central sentinel component of cellular metabolism that regulates the key aging processes including nutrient availability, energy homeostasis, cellular senescence, cell stemness, and proteostasis (22, 23). Although the expression of mTOR was not significantly associated with age (*p* = 0.353), there was an association between age and levels of ribosomal protein S6 kinase beta 1 (RPS6KB1) as a hallmark of activation by mTOR (*p* = 0.0001), which tended to increase with age (Figure 5A). Notably, expression of *RPS6KB1* was significantly elevated in the elderly subjects (60–79 years) when compared to those in the young group (aged 20–29 years). Furthermore, we verified whether mTOR-S6 kinase signaling module as the key aging-regulator is involved in gut barrier defense by analyzing the GTEx dataset. Expression of *mTOR* or *RPS6KB1* was associated with levels of gut defense biomarkers (Figures 5B,C). Subjects with high expression of *mTOR* or *RPS6KB1* presented low levels of *LYZ, CLDN3, PIGR*, and *LCN2* in the intestine, thereby indicating a negative regulation of gut defense by mTOR-S6 kinase signaling module. Since mTOR-S6 kinase signaling facilitates processes that fuel cell growth and proliferation, the signaling module counteracts cell differentiation to polarized enterocytes and other specialized intestinal epithelial cells such as goblet cells and Paneth cells, which is crucial for maintaining the gut epithelial barrier integrity (24). As a key intestinal differentiation factor, Krüppel-like factor 4 (*KLF4*) expression tended to decrease with age (Supplementary Figure 2D). Moreover, subjects with high expression of *mTOR* or *RPS6KB1* displayed low levels of *KLF4* in the intestine (Figures 5B,C), indicating insufficient differentiation and immature gut barrier by mTOR-S6 kinase signaling activation with age.

**Figure 6:**
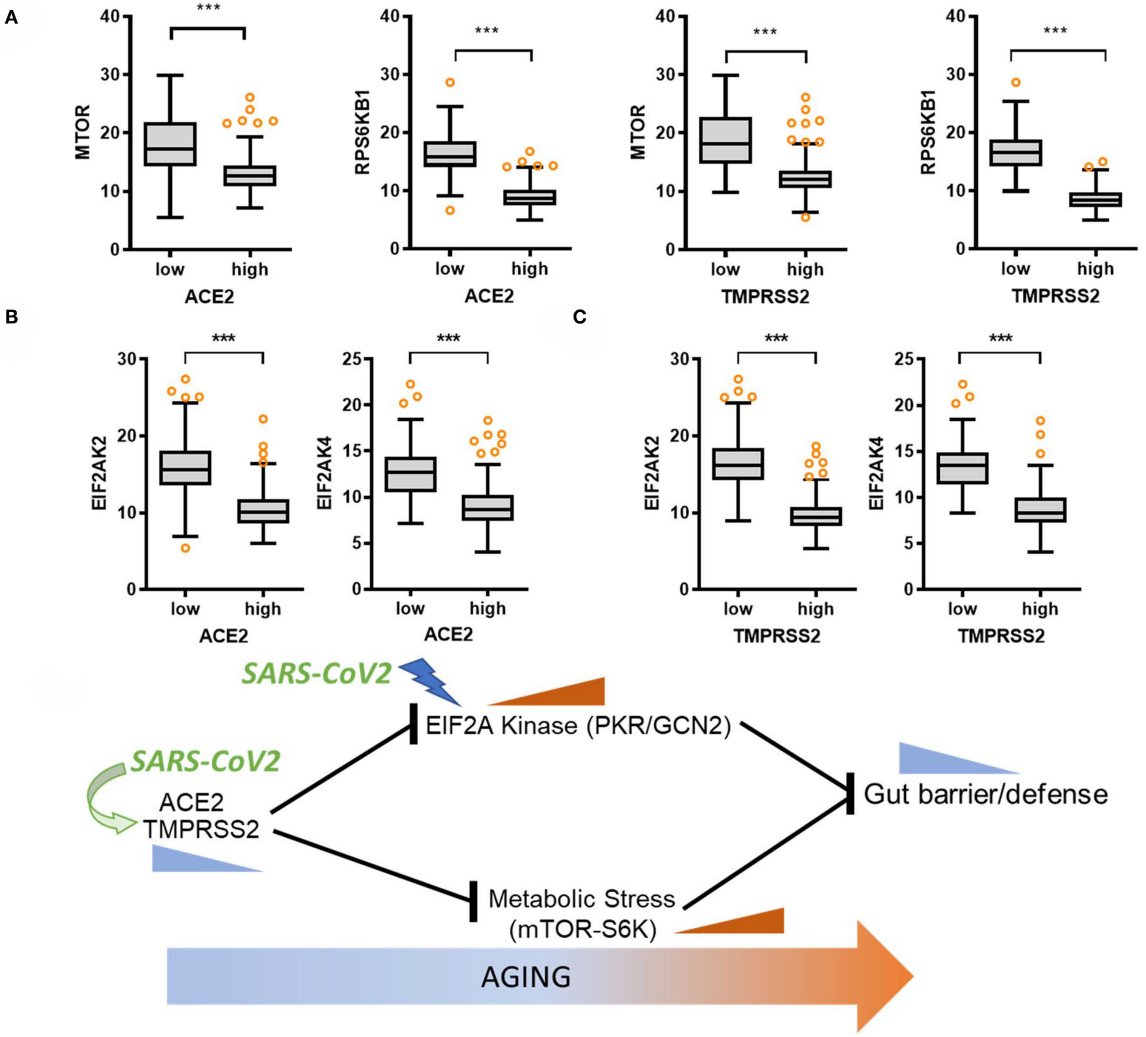
Regulation of stress responsive biomarkers by SARS-CoV-2 receptor. Considering the *ACE2* or *TMPRSS2* levels in normal mucosal intestinal tissues (GTEx dataset v8), we selected the 150 highest and 150 lowest level samples, which were further evaluated based on the expression of cell metabolic stress markers (**A**, *mTOR* or *RPS6KB1*) or eIF2α kinases (**B**, *EIF2AK2 or EIF2AK4*). Values are presented as transcripts per million (TPM). Asterisks (*) indicate significant differences from the low expression group (***p < 0.001). **(C)** A putative scheme for SARS-CoV-2 receptor-mediated modulation of gut barrier defense with age. In response to SARS-CoV-2 infection, SARS-CoV-2-responsive receptors positively contribute to maintenance of gut barrier defense via suppressing two stress signaling pathways including eIF2α kinase and mTOR-S6 kinase.

We studied two stress signaling modules (eIF2α kinase and mTOR-S6 kinase) counteracting gut barrier integrity via clinical transcriptome analysis. Moreover, eIF2α kinase and mTOR-S6 kinase signaling modules were assessed for their association with levels of SARS-CoV-2 receptors. Subjects with high expression of *ACE2* or *TMPRSS2* displayed low levels of *EIF2AK2, EIF2AK4, mTOR*, or *RPS6KB1* (Figures 6A,B), thereby supporting negative regulation of eIF2α kinase and mTOR-S6 kinase signaling by SARS-CoV-2 receptors in the gastrointestinal tract.

## Discussion

As a prediction model from the results, SARS-CoV-2-responsive receptors positively contribute to maintenance of gut barrier defense via attenuation of two stress signaling pathways of eIF2α kinase and mTOR-S6 kinase (Figure 6C). Nevertheless, the expression of viral receptors diminishes with age, thereby elevating two stress signaling modules and subsequently weakening the gut barrier defense in elderly subjects. The gut acts as an alternative source of SARS-CoV-2 infection, leading to symptoms such as diarrhea and prolonged fecal shedding of the virus, which potently occurs due to high levels of SARS-CoV-2 receptors in the gastrointestinal tract. High expression of *ACE2* in the intestinal epithelial cells implicates two potent routes of infection into the gastrointestinal tract. First, the well-known airway infection via human-to-human transmission presumably spreads via circulation to the rest of the body including gut and liver. The second route of gastrointestinal infection is airway-bypassing fecal–oral transmission from infected water or food. In particular, ACE2 acts as a coreceptor for nutrient uptake and particularly amino acid absorption from food (25), thereby indicating that SARS-CoV-2 in the contaminated food utilizes the receptor for its entry into the human body. Based on recent clinical evidences, ~50% of the COVID-19 patients present detectable levels of fecal SARS-CoV-2 RNA even after its clearance from the respiratory tract (11–13, 15, 25, 26), indicating that the digestive tract may act as a major site of viral replication and activity. Moreover, the infected gastrointestinal tract can be a crucial source of proinflammatory mediators such as bacterial products, metabolites, and gut-derived immune components which reversely aggravate the disease severity in the respiratory tract and other organs in infected hosts. This gut-to-airway infection supports the recent experimental evidence that intragastric inoculation of SARS-CoV-2 causes productive infection and leads to pulmonary pathological changes (27). Collectively, the enteric entry and replication of SARS-CoV-2 can be one of pivotal pathogenic pathways in addition to the airway infection.

Expression of the SARS-CoV-2 receptor is high in the gut; however, it decreases with age according to our transcriptomic analysis of the clinical dataset (Figure 1A). Nevertheless, the elderly subjects are more susceptible to COVID-19 than the younger groups in the recent global pandemic. In the present study, we propose mechanistic links of high disease severity in the elderly patients with low level of SARS-CoV-2 receptors. In addition to the SARS-CoV-2 receptors, the virus can impact host physiology via ISR. In case of SARS-CoV-2 infection, cells display EIF2AK2 (PKR)-linked pathogenesis including the ribosomal stress response via specific cleavage of 28S rRNA (19–21). Even though levels of the virus entry receptors decrease with age, the viral RNA triggers ribosomal stress leading to PKR activation and ISR via pattern recognition receptors, which can contribute to SARS-CoV-2-induced mucosal pathogenesis. Epithelial PKR activation plays a pivotal role in gut barrier disruption by regulating the lipid raft including caveolae (28). Moreover, lipid rafts contribute to SARS-CoV-2 infection in the early replication process (29, 30). Notably, ACE2 is located in the lipid rafts, which potently plays a pivotal role in the initial step of the virus entry-triggered signaling. PKR activation-induced structural alterations in lipid rafts facilitate caveolae-mediated degradation of epidermal growth factor receptor that is a crucial signaling mediator for maintaining the gut epithelial barrier integrity (28). PKR-linked molecular events during virus entry are well consistent with the patterns in clinical transcriptome analyses in the present study. Elevated levels of PKR signaling were associated with deterioration of gut defense with age despite attenuated *ACE2* expression in the elderly subjects. Therefore, ISR-linked disruption of gut defense may be an important mechanism of COVID-19 severity in elderly groups with low level of SARS-CoV-2 receptors.

We propose that mTOR-S6 kinase signaling is inversely associated with the expression of *KLF4*, a key intestinal differentiation factor. Due to insufficient cues for differentiation, formation of goblet and Paneth cells can be retarded in the gut barrier, which results in deficiencies in mucus and lysozyme secretion (24). Therefore, aging-associated increase of mTOR-S6 kinase signaling potently counteracts KLF4-mediated differentiation of the gut barrier cells, which can account for reduced mucosal defense against SARS-CoV-2 infection in the elderly population. Moreover, mTOR-S6 kinase signaling directly inhibits adenosine monophosphate-activated protein kinase (AMPK), the key regulator of energy metabolism, to promote cell proliferation under nutrient stress (31). Since AMPK improves gut epithelial differentiation and barrier function (32), mTOR may downregulate gut defense via attenuation of AMPK pathway. Mechanistically, AMPK inactivation is associated with reduced expression of caudal type homeobox 2 (*CDX2*), the key transcription factor for intestinal epithelium maturity and Paneth cell development (33). Detailed molecular epigenetic machinery of *CDX2* expression can be associated with polycomb repressive complex 2-regulated enrichment of H3K27me3 and lysine-specific histone demethylase-1-mediated reduction of H3K4me3 (32). Collectively, cell metabolic stress signaling of mTOR-S6 kinase potently attenuates AMPK activation, thus contributing to immature epithelial barrier via insufficient cellular differentiation with age. Although the expression of SARS-CoV-2 receptors is inversely associated with two stress signaling modules (eIF2α kinase and mTOR-S6 kinase), the levels of SARS-CoV-2 receptors diminish with age. Instead, elevated two stress signaling modules were positively involved in defective gut defense in the elderly subjects. Furthermore, disrupted gut barrier may increase the exposure to infectious agents and subsequently excessive inflammatory responses, which could be a crucial step of COVID-19 severity associated with age; however, extensive experimental evidences are still warranted to support the clinical transcriptome-based predictions of age-associated responses to COVID-19 and future interventions with the planet disorder.

## Data Availability Statement

The datasets generated for this study can be found in online repositories. The names of the repository/repositories and accession number(s) can be found in the article/Supplementary Material.

## Ethics Statement

Ethical review and approval was not required for the study on human participants in accordance with the local legislation and institutional requirements. Written informed consent for participation was not required for this study in accordance with the national legislation and the institutional requirements.

## Author Contributions

Project design and hypotheses were developed by YM. YM analyzed the data, prepared the manuscript, and supervised the overall project.

## Conflict of Interest

The author declares that the research was conducted in the absence of any commercial or financial relationships that could be construed as a potential conflict of interest.
