# Supplementary material for: Public Database-Driven Insights Into Aging Stress-Associated Defective Gut Barrier With Low SARS-CoV-2 Receptors
Source: Front Med (Lausanne). 2020 Dec 22;7:606991. doi: 10.3389/fmed.2020.606991 (PMC7783319; doi:10.3389/fmed.2020.606991)
Supplement: Supplementary file 1 [file Data_Sheet_1.PDF]

Supplementary Material

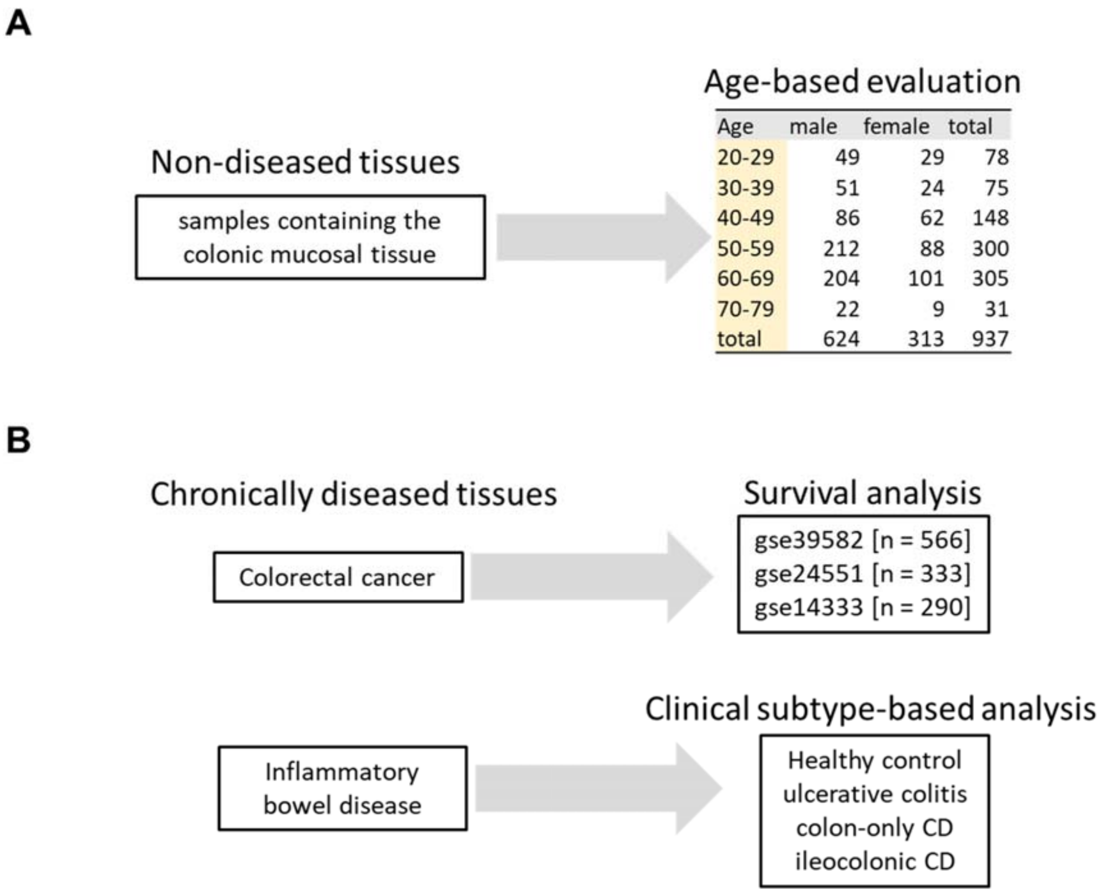

Supplementary Figure S1. Schematic diagram of the tissue analysis (A) Non-diseased tissue analysis in human population with age. (B) Evaluation of survival and gene expression profiles in patients with colorectal cancer or IBD.

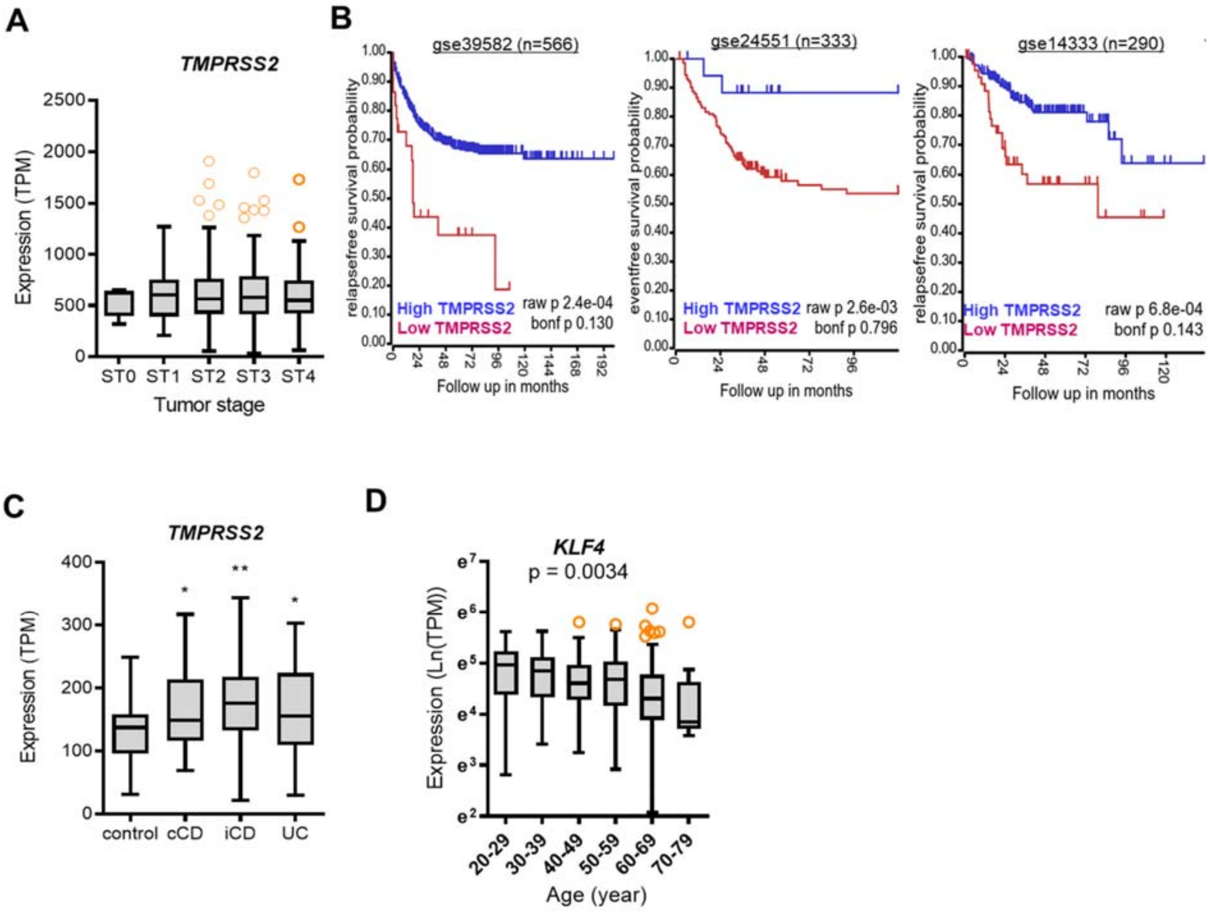

Supplementary Figure S2. (A) TMPRSS2 expression in different tumor stages from the transcriptome dataset in patients with colon cancer (GEO ID: gse39582, $n = 566$). Values are presented as natural logarithm of transcripts per million (TPM). Asterisks (*) indicate significant differences from levels in the stage 0 (*$p < 0.05$, **$p < 0.01$, ***$p < 0.001$ using two-tailed unpaired Student's *t*-test). (B) Kaplan–Meier plot of survival analysis based on tissue *TMPRSS2* transcript levels in patients with CRC from three datasets (gse39582 [$n = 566$, expression cutoff 72.2], gse24551 [$n = 333$, expression cutoff 300.0], and gse14333 [$n = 290$, expression cutoff 267.8]). (C) Intestinal expression of TMPRSS2 was assessed in patients with different IBD types from datasets gse117993 ($n = 190$). UC, ulcerative colitis; cCD, colon-only CD; iCD, ileocolonic CD. Values are presented as transcripts per million (TPM). Results are depicted as box-and-whisker plots (Turkey). Asterisks (*) indicate significant differences from the control group (*$p < 0.05$, **$p < 0.01$, ***$p < 0.001$ using two-tailed unpaired Student's *t*-test). (D) Results are depicted as box-and-whisker plots (Turkey) for the expression of *KLF4* in normal mucosal intestinal tissues (GTEx dataset v8). Values are presented as natural logarithm of transcripts per million (TPM). Statistical significance of the expression variation with age is illustrated on the top of each plot (Kruskal–Wallis test).
